# *De novo* Transcriptome Assembly and the Putative Biosynthetic Pathway of Steroidal Sapogenins of *Dioscorea composita*

**DOI:** 10.1371/journal.pone.0124560

**Published:** 2015-04-10

**Authors:** Xia Wang, Dijia Chen, Yuqi Wang, Jun Xie

**Affiliations:** 1 Institute of New Energy and New Materials, South China Agriculture University, Guangzhou, 510642, P. R. China; 2 Key Laboratory of Biomass Energy of Guangdong Regular Higher Education Institutions, Guangzhou, 510642, P. R. China; 3 Key Laboratory of Energy Plants Resource and Utilization, Ministry of Agriculture, P. R. China, Guangzhou, 510642, P. R. China; The National Orchid Conservation Center of China; The Orchid Conservation & Research Center of Shenzhen, CHINA

## Abstract

The plant *Dioscorea composita* has important applications in the medical and energy industries, and can be used for the extraction of steroidal sapogenins (important raw materials for the synthesis of steroidal drugs) and bioethanol production. However, little is known at the genetic level about how sapogenins are biosynthesized in this plant. Using Illumina deep sequencing, 62,341 unigenes were obtained by assembling its transcriptome, and 27,720 unigenes were annotated. Of these, 8,022 unigenes were mapped to 243 specific pathways, and 531 unigenes were identified to be involved in 24 secondary metabolic pathways. 35 enzymes, which were encoded by 79 unigenes, were related to the biosynthesis of steroidal sapogenins in this transcriptome database, covering almost all the nodes in the steroidal pathway. The results of real-time PCR experiments on ten related transcripts (*HMGR*, *MK*, *SQLE*, *FPPS*, *DXS*, *CAS*, *HMED*, *CYP51*, *DHCR7*, and *DHCR24*) indicated that sapogenins were mainly biosynthesized by the mevalonate pathway. The expression of these ten transcripts in the tuber and leaves was found to be much higher than in the stem. Also, expression in the shoots was low. The nucleotide and protein sequences and conserved domains of four related genes (*HMGR*, *CAS*, *SQS*, and *SMT1*) were highly conserved between *D*. *composita* and *D*. *zingiberensis*; but expression of these four genes is greater in *D*. *composita*. However, there is no expression of these key enzymes in potato and no steroidal sapogenins are synthesized.

## Background

Steroidal sapogenins exist naturally as aglycones of steroidal saponins (a large family with enormous structural diversity) which have attracted a great deal of interest lately due to their wide spectrum of biological and pharmacological functions [1–2]. Steroidal saponins are among the more widely distributed secondary metabolites within the plant kingdom [3]. Saponin-deficient mutants have their defenses compromised to a range of pathogens, confirming the saponins’ roles in plant resistance [4]. Steroidal saponins have been a significant ingredient in traditional medicine for a long time to treat cardiac disease, cancer, fungal infection, and so on[5–6].

In the medical industry, steroidal sapogenins are widely used as the starting material for the synthesis of many steroidal drugs (e.g. antioxidants, anti-inflammatories, androgen, estrogen, and contraceptives) due to the similarity between their skeletons and those of the steroidal medicine [7]. More importantly, steroidal sapogenins are attractive to many synthetic and medicinal chemists aiming to harness their anticancer activity [8].

The major steroidal sapogenins exist mainly in monocots such as some species of the Dioscoreaceae family [9]. *Dioscorea composita* is one of the most important resources because its tuber is rich in steroidal sapogenins and starch. *D*. *composita* originates from Mexico and is suited to tropical and subtropical zones [10]. In 1980, *D*. *composita* was successfully imported and planted in China for the first time by the Xishuangbanna Tropical Botanical Garden, Chinese Academy of Sciences [11]. According to an assay we undertook, the steroidal sapogenin content in the tuber of *D*. *composita* was 3.68% ± 0.5% (dry weight), which was approximately twice that of *Dioscorea zingiberensis* (the primary material used to extract sapogenins in China for several decades). The harvest of *D*. *composita* obtained was 45–90 tons per hectare, much higher than that for *D*. *zingiberensis*. For these reasons, *D*. *composita* appears to be an excellent medicinal and energy-resource providing plant.

**Fig 1:**
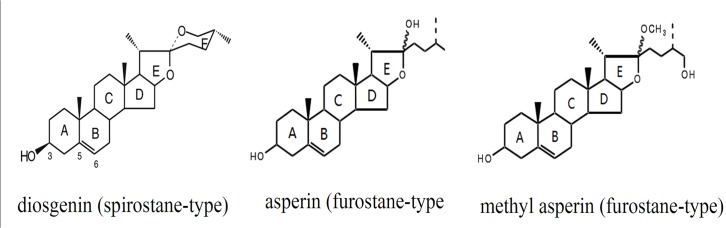
The structures of some of the steroidal sapogenins in *D*. *composita*.

The major steroidal sapogenins isolated from *D*. *composita* can be divided into spirostane- and furostane-type sapogenins according to the structures of their skeletons [12]. The main compound, diosgenin, belongs to the spirostane-type and has a hexacyclic ABCDEF-ring system (Fig 1). Two other important ones, asperin and methyl asperin, belong to the furostane-type and possess ABCDE-ring systems [13].

Steroidal sapogenins are complex molecules and are synthesized by equally complex biosynthetic pathways. As there is no genetic information about *D*. *composita*, it is very difficult to reveal the biosynthesis of the active ingredients. Recently, great advances in transcriptome deep sequencing technology and bioinformatics have opened a new avenue for such investigations. Compared with the conventional methods for gene cloning and sequencing, the new generation sequencing technology affords a quick, easy, and full-scale method of investigation, especially in the context of metabolism research. The technique has already been used to investigate the transcriptome of various plants, e.g. *Salvia miltiorrhiza*[14], *Fagopyrum esculentum* [15], *Craterostigma plantagineum* [16], and others. In this article, the Illumina deep RNA sequencing (RNA-Seq) method was used to analyze the transcriptome of 18-month-old *D*. *composita* at harvest. Using the technique, a list of putative genes involved in the biosynthetic pathway of diosgenin were determined. The results are helpful for enriching the gene resources of *D*. *composita* and discovering the genes related to valuable ingredients. They also contribute to enhancing the active components by metabolic regulation (or other biological methods) on the basis of the transcriptome database of *D*. *composita*.

## Results and Discussion

### Short-reads *de novo* sequencing and assembly

Illumina deep RNA sequencing technology was employed to sequence the transcriptome of *D*. *composita*. Samples were collected from six plants for conducting RNA-Seq analysis. In total, 114,136,043 clean paired-end reads were obtained corresponding overall to 8.0 Gbp (NCBI accession: SRR1525775, SRR1525776, SRR1525777, SRR1525779, SRR1525790, SRR1525791, SRR1525792, and SRR1525793). All of these clean paired-end reads were pooled together and *de novo* assembled by using Trinity software (v2012-10-15) [17]. Finally, 131,655 transcripts with length above 200 bp were obtained. The average length of these transcripts was 1,368 bp and the N50 length was 2,303 bp. There were 62,285 transcripts with length ≥1,000 bp, 31,868 transcripts with length ≥ 2,000 bp, and the max length was 25,464 bp. The longest transcript of each gene was selected as the unigene, and we generated 62,341 unigenes above 200 bp. The average length of these unigenes was 724 bp, and the N50 length was 1,228 bp. There were 14,425 unigenes ≥ 1,000 bp, 5,975 unigenes ≥ 2,000 bp, and the max length was 22,744 bp (Table 1). Common perl scripts were used to conduct the statistical analysis.

**Table 1:** Summary of the transcript statistics generated from *D*. *composita*.

Item	Characteristics
Number of paired-end reads	114,136,043
Transcript number	131,655
Transcripts ≥ 1000 bp	62,285
Transcripts ≥ 2000 bp	31,868
Average length of transcripts (bp)	1,368
Max length of transcripts (bp)	25,464
N50 length of transcripts (bp)	2,303
Unigene number	62,341
Unigenes ≥ 1000 bp	14,425
Unigenes ≥ 2000 bp	5,975
Average length of Unigenes (bp)	724
Max length of Unigenes (bp)	22,744
N50 length of Unigenes (bp)	1,228

To evaluate the final assembly, the ratios of the conserved domain sequences (CDSs) with different lengths containing transcripts to the total number of transcripts were calculated. For example, there were 17,346 transcripts containing long-CDSs with length ≥ 1,500 bp, so the ratio was 13.2% (compared with 131,655, the total number of transcripts—see Table 2). In the final results, 83,114 transcripts contained CDSs with length ≥ 300 bp (a ratio of 63.1%).

**Table 2:** Summary of the statistics of the CDS-containing sequences.

Length of CDS (bp)	Number of transcripts	Ratio (%)
300–599	27,398	20.8
600–899	15,368	11.7
900–1199	13,797	10.5
1200–1499	9,205	7.0
≥ 1500	17,346	13.2
Total	83,114	63.1

Additionally, the authors aligned the 396 nucleotide sequences of *Dioscorea* from the National Center for Biotechnology Information (NCBI) with the total transcripts under the condition that the blast length was greater than 200 bp. The number of sequences hit was 370 with an average sensitivity of 81.5%. These assessments suggest that the assembly was satisfactory.

### Functional annotation and classification of *D*. *composita* transcriptome

To investigate the function of these unigenes, sequence similarity was compared with several databases: the non-redundant protein database (NR) and the Arabidopsis protein database and the Rice protein database using Blastx software (e values < 10^–5^)[18], the Kyoto Encyclopedia of Genes and Genomes (KEGG) database using KAAS software (e values < 10^–10^)[19]. The protein family and domain predictions were completed by using the protein family (Pfam) database andhmmscan software package(e values < 10^–2^)[20]. A total of 27,720 unigenes (44.5%) were annotated using the public protein databases (see Table 3 and S1–S5 Tables).

**Table 3:** Summary of the functional annotation statistics for the *D*. *composita* unigenes in the protein databases.

Annotated database	Number of unigene	Percentage (%)
NR	22,815	36.6
Arabidopsis protein database	20,257	32.5
Rice protein database	21,017	33.7
KEGG	8,022	12.9
Pfam	20,747	33.3
Total	27,720	44.5

**Fig 2:**
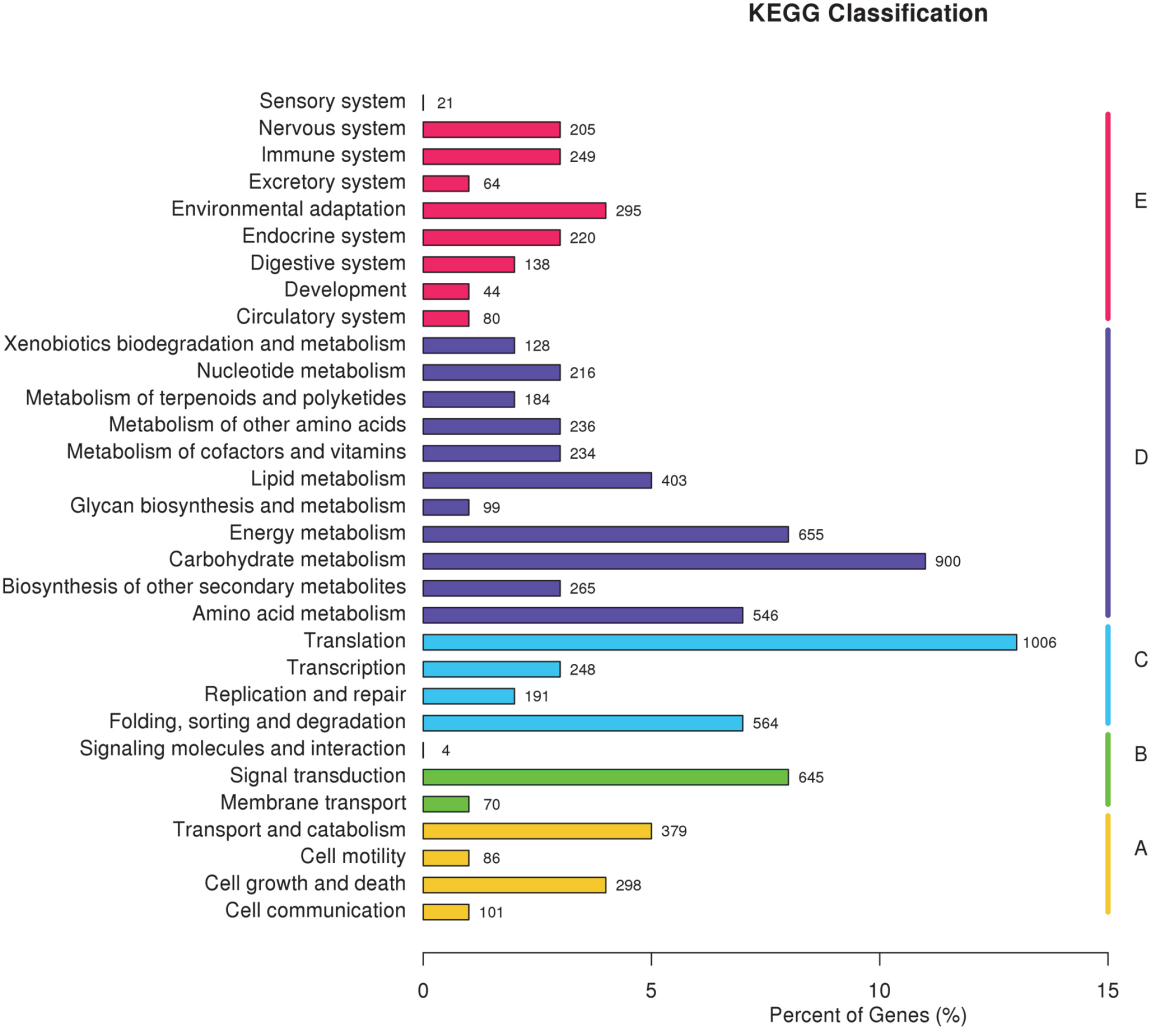
KEGG classification of the unigenes from RNA-Seq experiments on *D*. *composita*. Key: A–cellular processes, B–environmental information processing, C–genetic information processing, D–metabolism, and E–organism systems.

KEGG classification of the annotated unigenes was executed. The results indicated that 8,022 (12.9%) of the unigenes could be mapped to the KEGG database. The analysis revealed that: 864 (10.8%) of these unigenes are clustered into a ‘cellular processes’ section wherein the majority of the terms relate to ‘transport and catabolism’; 719 (9.0%) are grouped into an ‘environmental information processing’ category and mostly enriched in ‘signal transduction’; 2,009 (25.0%) of the unigenes are clustered in a ‘genetic information processing’ section in which more than half belong to a ‘translation’ sub-section; 3,866 (48.2%) are clustered into a ‘metabolism’ category and the majority of these relate to ‘carbohydrate metabolism’; 1,316 (16.4%) of the unigenes can be grouped into a ‘organism systems’ category, where the largest sub-section is ‘environmental adaptation’ (see Fig 2).

### Secondary metabolic pathway analysis

**Fig 3:**
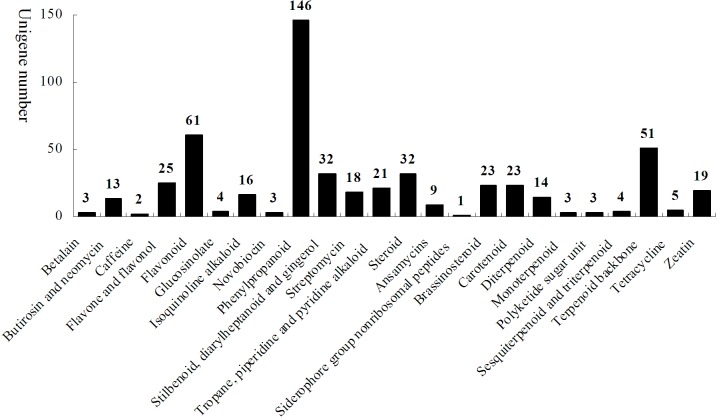
Unigenes related to secondary metabolism from RNA-Seq experiments on *D*. *composita*.

KEGG maps provide much information to help understand the high-level functions of a biological system. Metabolic pathway analysis enables us to realize which genes in the same pathway cooperate with each other as they exercise their biological functions. In all, 8,022 unigenes could be mapped with 243 metabolic pathways based on the KEGG blast analysis, and 531 unigenes were mapped into 24 secondary metabolic biosynthetic pathways (Fig 3). These secondary metabolic genes supplied further information for analyzing the secondary metabolite biosyntheses. Many unigenes which encode key enzymes in the biosynthetic pathways for phenylpropanoids, flavonoids, steroids, stilbenoid, diarylheptanoid and gingerol in *D*. *composita* were confirmed.

### Steroidal sapogenin biosynthetic pathways

**Fig 4:**
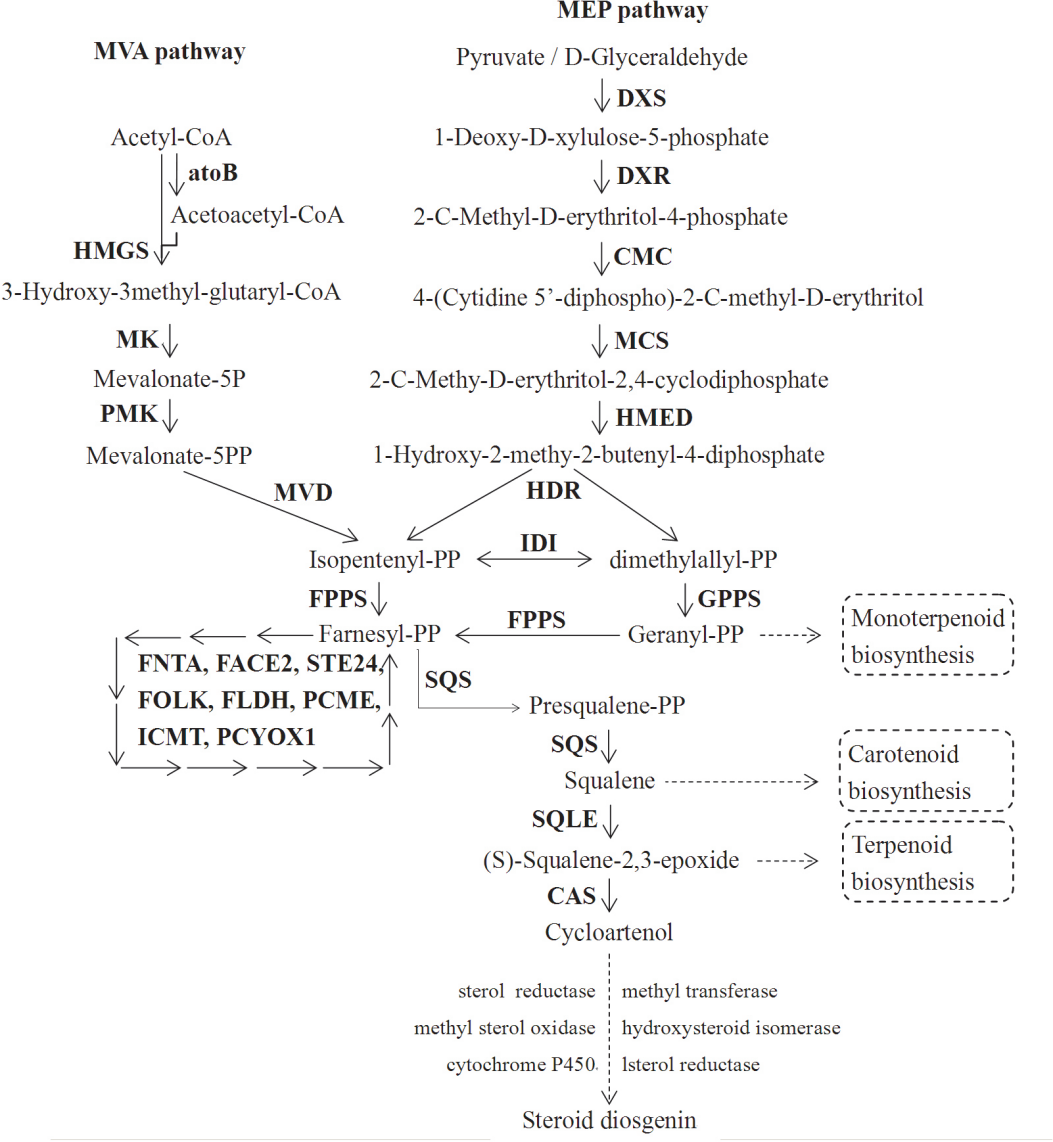
Putative biosynthetic pathway of steroidal sapogenin in *D*. *composita*. Key: atoB: acetyl-CoA C-acetyltransferase, HMGS: hydroxymethylglutaryl-CoA synthase, HMGR: 3-hydroxy-3-methylglutaryl-CoA reductase, MK: mevalonate kinase, PMK: phosephomevalonate kinase, MVD: diphosphomevalonate decarboxylase, DXS: 1-deoxy-D-xylulose-5-phosphate synthase, DXR: 1-deoxy-D-xylulose-5-phosphate reductoisomerase, CMC: 2-C-methyl-D-erythritol 4-phosphate cytidylyltransferase, CMK: 4-diphosphocytidyl-2-C-methyl-D-erythritol kinase, MCS: 2-C-methyl-D-erythritol 2,4-cyclodiphosphate synthase, HMED: 4-Hydroxy-3-methy-2-butenyl-diphosphate synthase, HDR: 1-Hydroxy-2-methy-2-butenyl-4-diphosphate reductase, IDI: isopentenyl diphosphate isomerase, GPPS: geranyl diphosphate synthase, FPPS: farnesyl diphosphate synthase, FNTA: protein farnesyltransferase, FACE2: prenyl protein peptidase, STE24: endopeptidase, FOLK: farnesol kinase, FLDH: farnesol dehydrogenase, PCME: prenylcysteine alpha-carboxyl methylesterase, ICMT: protein-S-isoprenylcysteine O-methyltransferase, PCYOX1: prenylcysteine oxidase, SQS: squalene synthase, SQLE: squalene monooxygenase, CAS: cycloartenol synthase.

Steroidal sapogenins in plants were biosynthesized from two common C_5_ isoprene unites: isopentenyl diphosphate and dimethylallyl diphosphate. These unites were synthesized by the cytosolic mevalonate (MVA) and plastial 2-C-methl-D-erythritol-4-phospate (MEP) pathways [21]. The MVA pathway played a major role in the production of steroidal backbones [22]. A total of 35 enzymes encoded by 79 unigenes were related to steroidal sapogenin biosynthesis in this transcriptome database (Table 4 and S6 Table). These almost cover the entire pathway. The protein families in which these unigenes are involved are listed in Table 4, the similarity of these unigenes with *Arabidopsis* and rice is presented in Table 5 and the primary biosynthetic pathway is illustrated in Fig 4.

**Table 4:** The number of unigenes (NU) potentially related to steroidal sapogenin biosynthesis from RNA-Seq experiments on *D*. *composita* and their ID.

Pathway	Enzyme	NU	Pfam ID
Terpenoid backbone	atoB	3	PF00108, PF02803
	HMGS	8	PF01154, PF08540, PF08541, PF08545, PF00560
	HMGR	6	PF03884, PF00368
	MK	1	—-
	PMK	1	PF00288
	MVD	1	PF00288
	IDI	2	PF00293
	GPPS	7	PF00348, PF00420, PF11057, PF04847, PF01690
	FPPS	1	PF08091, PF00348
	FNTA	1	PF01239
	FACE2	1	PF02517
	STE24	1	PF01435
	FOLK	1	PF01254, PF01148
	FLDH	1	PF01370, PF04321, PF02719, PF00106, PF01073
	PCME	1	PF02230, PF01738, PF07859, PF00326, PF01179
	ICMT	1	PF04140
	PCYOX1	1	PF07992, PF01593, PF01266
	DXS	4	PF02780, PF00676, PF02775, PF04805, PF07817
	DXR	1	PF03447, PF02670, PF08436, PF01118
	CMK	1	—-
	MCS	1	PF02542
	HMED	1	PF05699, PF04551, PF00293, PF05026, PF00118
	HDR	1	PF00643, PF01667, PF01485
Sesquiterpenoid and triterpenoid biosynthesis	SQS	1	PF00494
	SQLE	1	PF01210, PF02737, PF05834, PF01266, PF08491, PF02558, PF07992, PF00070, PF01134
Steroid biosynthesis	CAS	1	PF04159, PF00432, PF07678
	Cycloeucalenol cycloisomerase	1	—-
	Sterol methyl oxidase	8	PF04116
	Methyl transferase	2	PF01135, PF08003, PF08241, PF08498, PF01209, PF02353, PF05175, PF02322, PF09445
	Carboxylate-dehydrogenase	2	PF01370, PF02719, PF04321, PF01073, PF02453
	Cytochrome P450	6	PF00067, PF00283,PF11023, PF01254,
	Hydroxysteroid-isomerases	1	PF04116, PF01545, PF00172,PF05241
	Sterol reductase	6	PF00014, PF01222, PF02326, PF01565, PF00424, PF02148, PF04790, PF06363, PF09088
	Methylsterol monooxygenase	2	PF04116
	Sterol desaturase	1	PF04116, PF01545, PF00172
Total	35	79	

**Table 5:** The similarity of unigenes (NU) potentially related to steroidal sapogenin biosynthesison *D*. *composita* with *Arabidopsis* and Rice.

Enzyme	Similarity ID	
	*Arabidopsis*	Rice
atoB	AT5G48230.1, AT5G47720.1	LOC_Os01g02020.3
HMGS	AT4G11820.1, AT4G11820.2	LOC_Os03g02710.1, LOC_Os08g43170.1, LOC_Os09g34960.1
HMGR	AT2G17370.1	LOC_Os09g31970.1
MK	AT5G27450.3	LOC_Os10g18220.1
PMK	AT1G31910.1	LOC_Os03g48160.1
MVD	—-	—-
IDI	AT3G02780.1	LOC_Os07g36190.1
GPPS	AT2G34630.2	LOC_Os06g46450.1
FPPS	AT5G47770.1	LOC_Os01g50760.1
FNTA	AT3G59380.1	LOC_Os09g33930.3
FACE2	AT2G36305.1	LOC_Os05g28950.1
STE24	AT4G01320.1	LOC_Os02g45650.1
FOLK	AT5G58560.1	LOC_Os01g61560.1
FLDH	AT4G33360.1	LOC_Os03g08624.1
PCME	AT5G15860.1	LOC_Os06g49440.1
ICMT	AT5G23320.1	LOC_Os04g51380.1
PCYOX1	AT5G63910.1	LOC_Os04g59630.1
DXS	AT4G15560.1, AT5G11380.2	LOC_Os05g33840.1, LOC_Os06g05100.3
DXR	AT5G62790.1	LOC_Os01g01710.1
CMK	—-	—-
MCS	AT4G25720.2	LOC_Os06g01410.1
HMED	AT5G60600.1	LOC_Os02g39160.1
HDR	AT4G34350.1	LOC_Os03g52170.1
SQS	AT3G59380.1	LOC_Os09g33930.3
SQLE	AT1G58440.1	LOC_Os03g12910.1
CAS	AT2G07050.1	LOC_Os02g04710.1
Sterol methyl oxidase	AT1G07420.1,AT1G07420.2,AT4G22753.1, AT2G29390.3,AT2G29390.4,AT2G29390.2,AT4G12110.1	LOC_Os11g48020.1, LOC_Os07g01150.3, LOC_Os10g39810.1
Methyl transferase	AT5G13710.2,AT1G20330.1	LOC_Os07g10600.2, LOC_Os03g04340.1
Carboxylate-dehydrogenase	AT2G43420.1,AT2G26260.1	LOC_Os09g34090.1, LOC_Os03g29170.1
Cytochrome P450	AT2G28860.1,AT2G34500.1,AT2G34490.1,AT1G11680.1	LOC_Os01g11300.1, LOC_Os01g11340.1, LOC_Os01g11270.1, LOC_Os11g32240.1
Hydroxysteroid- isomerases	AT3G02580.1,AT1G20050.1	LOC_Os01g04260.1, LOC_Os01g01369.1
Sterol reductase	AT3G19820.3, AT3G52940.1,AT1G50430.1	LOC_Os10g25780.1,LOC_Os10g25780.3, LOC_Os09g39220.1, LOC_Os02g26650.3
Cycloeucalenol cycloisomerase	AT5G50375.1	LOC_Os11g19700.1
Methylsterol monooxygenase	AT4G12110.1	LOC_Os10g39810.1
Sterol desaturase	AT3G02580.1	LOC_Os01g04260.1

As shown in Fig 4, the complete biosynthetic pathway was divided into three parts according to the KEGG classification of this transcriptome: a steroidal backbone part, a sesquiterpenoid and triterpenoid part, and a steroid biosynthesis part. In the first part, the steroidal sapogenin backbones were biosynthesized from acetyl-CoA (C2) to farnesyl diphosphate (C15) in 17 steps which involved 23 enzymes and 47 unigenes. Farnesyl diphosphate was formed by dimethylallyl diphosphate and two isopentenyl diphosphate molecules in a sequential head-to-tail condensation. This process was the same as in terpenoid sapogenin synthesis. Within the sesquiterpenoid and triterpenoid biosynthesis section, two farnesyl diphosphate molecules were condensed tail-to-tail style [21]. This was followed by oxidation to form squalene-2,3-epoxide (C30) in 3 steps—2enzymes and 2 unigenes participate in these steps. In contrast to terpenoid sapogenin biosynthesis, squalene-2,3-epoxide transformed to a chair-boat-chair-boat conformation but not a chair-chair-chair-boat one. In the steroidal biosynthesis part, squalene-2,3-epoxide with chair-boat-chair-boat structure was cycled to cycloartenol (C30) which was regarded as the precursor to the steroidal sapogenins [23–25]. Then, the steroidal sapogenins (C28) which are produced underwent various modifications through the action of many enzymes, e.g. cytochrome P450, oxidase, reductase, isomerase, transferase, etc [26]. However, the specific steps involved in steroidal sapogenin biosynthesis from cycloartenol were unclear in the plants of the Dioscoreaceae family.

### Expression profiles of 10 transcripts related to the biosynthesis of steroidal sapogenin

**Fig 5:**
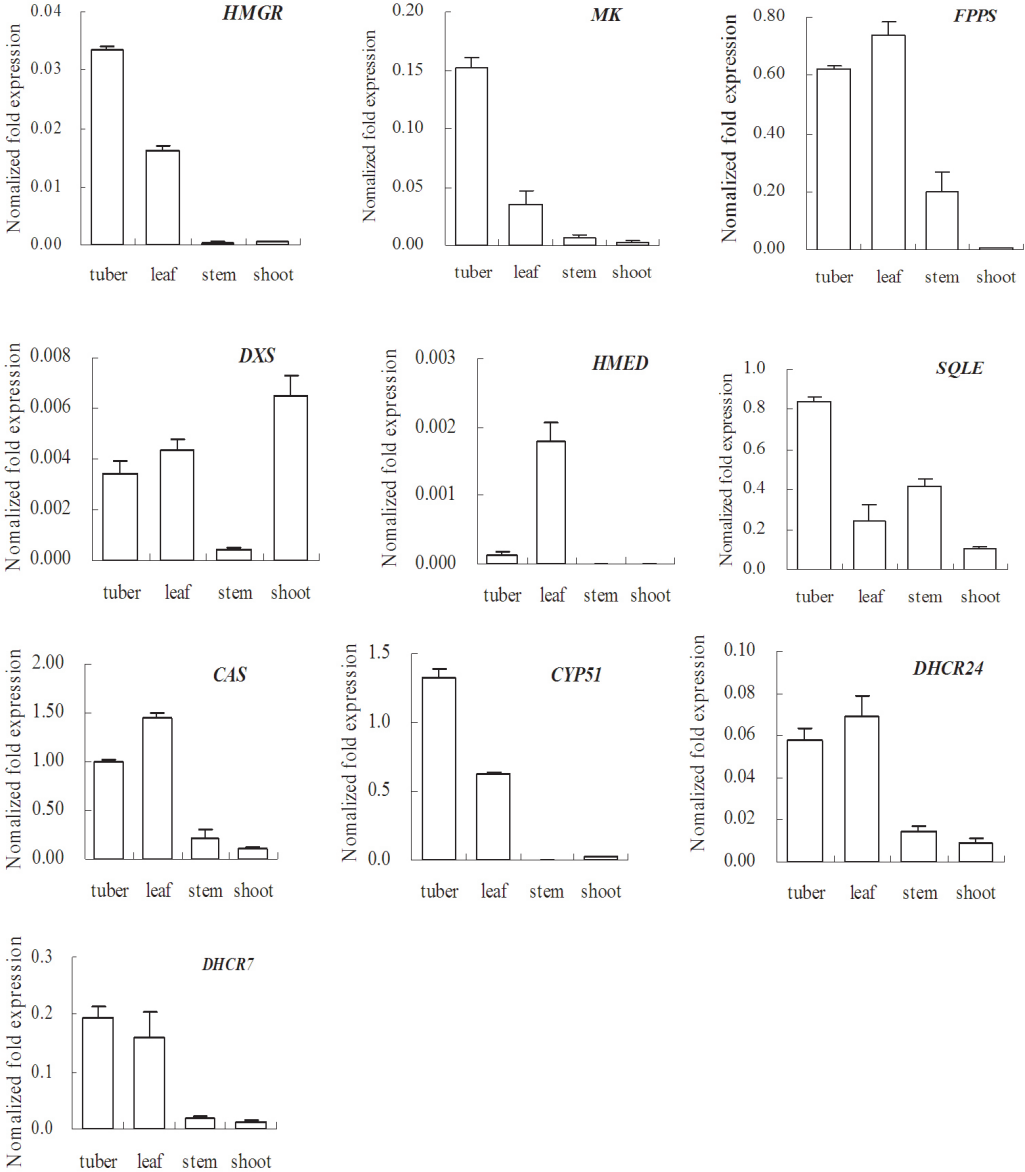
Expression patterns for ten transcripts related to steroidal sapogenin in *D*. *composita*. The tuber, leaf, and stem results relate to plants that are 18-month-old. The shoots results relate to 1-month-old plants. (Key: CYP51: sterol 14-demethylase, DHCR24: delta 24-sterol reductase, DHCR7: 7-dehydrocholesterol reductase.)

*D*. *composita* tuber was used as raw material to extract sapogenin. It is interesting to discover whether or not related gene expression is correlated with developmental stages and tissues. The authors used relative quantitative RT-PCR to analyze the expression profiles of 10 transcripts related to steroidal sapogenin biosynthesis using material from the leaves, stems, and tubers of 18-month-old plants. The results are shown in Fig 5. Results show that all the genes selected, except *HMED* and *CYP51*, were expressed in the tuber, leaf, and stem of the plant. Five genes (*HMGR*, *MK*, *SQLE*, *CYP51*, and *DHCR7*) exhibited their largest expression levels in the tuber. The other five genes (*FPPS*, *DXS*, *HMED*, *CAS*, and *DHCR24*) had larger expression levels in the leaves. The expression levels in the tubers and leaves of all the genes selected were much higher than in the stems. This result reveals that the plants produced a variety of compounds by this pathway and their functions were different. Some of the products related to membrane components, e.g. phytosterols, so the related genes were expressed in all tissues [27]. Other compounds, like sapogenins, functioned to counteract pathogens and herbivores, and were mainly biosynthesized and stored in the tubers. The leaves were able to metabolize the triterpenic constituent of leaf wax in their defensive glands [28]. The expression of the genes involved in the MVA pathway (*HMGR* and *MK*) was much greater than the genes involved in the MEP pathway (*DXS* and *HMED*). This difference between the two pathways indicated that the biosynthesis of steroidal sapogenins chiefly occurred via the MVA pathway in *D*. *composita*.

Our study also compared the expression patterns in 1-month-old shoots as well (Fig 5). Compared to 18-month-old plants, 9 genes (all except *DXS*) were expressed in 1-month-old shoots at much lower levels. These results reveal that the secondary metabolism was not prevalent at the seedling stage which suggests the saponins might not be required for seedling development and survival [29]. The expression of *DXS* in shoots was higher than in all organs of the 18-month-old plants, which indicates that *DXS* played a role in producing gibberellins which improved the growth of leaves and budlets [15].

### The differences between *D*. *composita* and other tuberous plants

To investigate why steroidal sapogenins are synthesized in *D*. *composita* more copiously than in other tuberous plants, the gene sequences, protein sequences, conserved domains, and expression quantities of steroids in several tuberous plants were analyzed.

### Steroidal sapogenins content

**Fig 6:**
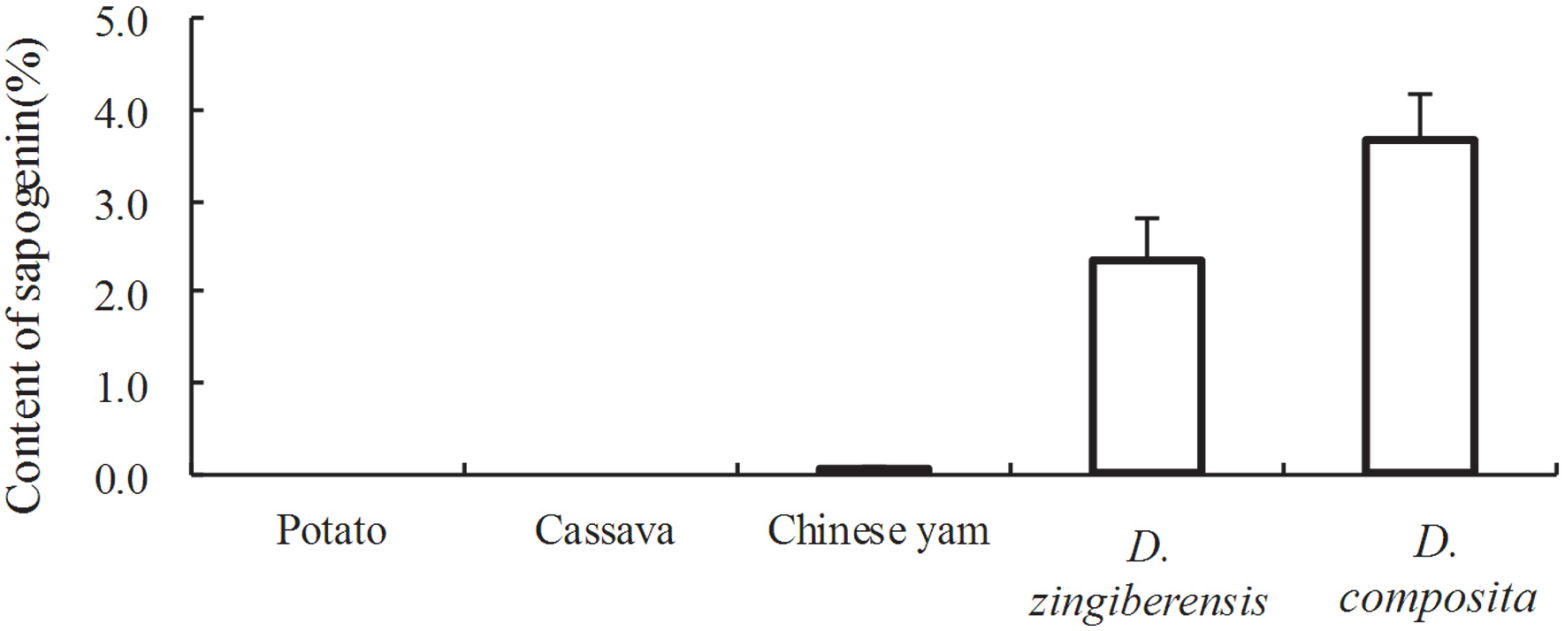
Content of steroidal sapogenins in potato, cassava, Chinese yam, *D*. *zingiberensis* and *D*. *composita* (dry weight).

The contents of steroidal sapogenins in *D*. *zingiberensis*, Chinese yam (*Dioscorea opposita* Thunb.), potato (*Solanum tuberosum* L.), and cassava (*Manihot esculenta*) were compared with that in *D*. *composita* (Fig 6). According to the measurements, the steroidal sapogenins content in *D*. *composita* was 1.56 times and 52.6 times that of *D*. *zingiberensis* and Chinese yam, respectively. There were no steroidal sapogenins determined in potato and cassava.

### Nucleotide sequences, protein sequences, and conserved domains

Four steroidal sapogenin synthesis-related enzymes: *HMGR*, *CAS*, *SQS* and *SMT1* (sterol 24-C-methyltransferase) were chosen for comparison in *D*. *composita*, *D*. *zingiberensis*, and potato (there was no relevant data published for Chinese yam and cassava). The results are shown in Tables 6–8. *HMGR* was involved in a rate-limiting step in the isoprene biosynthesis via the MVA pathway. *CAS* was a key enzyme in steroidal sapogenin precursor synthesis. *SQS* produced the triterpene and tetraterpene precursors for many diverse sterol and carotenoid end products. *SMT1* was ‘in charge’ of protein structure modification. All the blast analyses were completed online through the NCBI website, and the conserved domains of *HMGR*, *CAS*, *SQS*, and *SMT1* in *D*. *composita* were calculated online using CDD v3.11–45746 PSSMs on the NCBI site.

**Table 6:** The identity of nucleotide sequences in *D*. *composita*, *D*. *zingiberensis*, and potato.

Gene	Plant	Accession	Length (bp)	Query cover	Identity
*HMGR*	*D*. *composita*		2409	–	–
	*D*. *zingiberensis*	KC960674.1	2077	89%	90%
	Potato	L01400.1	2379	No	No
*CAS*	*D*. *composita*		3227	–	–
	*D*. *zingiberensis*	AM697885.1	2703	84%	89%
	Potato	XM_006362747.1	2616	No	No
*SQS*	*D*. *composita*		1825	–	–
	*D*. *zingiberensis*	KC960673.1	1453	86%	89%
	Potato	JF802610.1	1236	No	No
*SMT1*	*D*. *composita*		5718	–	–
	*D*. *zingiberensis*	FR714840.1	1459	68%	89%
	Potato	XM_006364446.1	1372	No	No

**Table 7:** The identity of protein sequences in *D*. *composita*, *D*. *zingiberensis*, and potato.

Protein	Plant	Accession	Length (aa)	Query cover	Identity
*HMGR*	*D*. *composita*		580	–	–
	*D*. *zingiberensis*	AGN32411.1	585	96%	92%
	Potato	AAA93498.1	596	100%	66%
*CAS*	*D*. *composita*		759	–	–
	*D*. *zingiberensis*	CAM91422.1	759	100%	90%
	Potato	XP_006362809.1	757	99%	77%
*SQS*	*D*. *composita*		408	–	–
	*D*. *zingiberensis*	AGN32410.1	409	95%	91%
	Potato	AEX26929.1	411	94%	76%
*SMT1*	*D*. *composita*		345	–	–
	*D*. *zingiberensis*	CBX33151.1	333	99%	93%
	Potato	XP_006364508.1	358	89%	40%

**Table 8:** The comparison of conserved domains in *D*. *composita*, *D*. *zingiberensis*, and potato.

Protein	Name of conserved domains	Plant	Identity
*HMGR*	HMG-CoA_reductase_classI	*D*. *composita*	–
		*D*. *zingiberensis*	93%
		Potato	82%
*CAS*	SQCY_1	*D*. *composita*	–
		*D*. *zingiberensis*	90%
		Potato	78%
*SQS*	Trans_IPPS_HH	*D*. *composita*	–
		*D*. *zingiberensis*	94%
		Potato	84%
*SMT1*	Sterol_MT_C	*D*. *composita*	–
		*D*. *zingiberensis*	79%
		Potato	30%

Key: HMG-CoA_reductase_classI: Class I hydroxymethylglutaryl-coenzyme A (HMG-CoA) reductase, accession: cd00643; SQCY_1: Squalene cyclase domain subgroup 1, accession: cd02892; Trans_IPPS_HH: Trans-Isoprenyl Diphosphate Synthases, accession: cd00683; Sterol_MT_C: Sterol methyltransferase C-terminal, accession: pfam08498.

For *HMGR*, *CAS*, *SQS*, and *SMT1*, the gene sequences (above 89%), protein sequences (above 90%), and conserved domains (above 79%) were similar in *D*. *composita* and *D*. *zingiberensis*. This suggests that the four enzymes were produced with quite consistent structure and function in closely related plants like *D*. *composita* and *D*. *zingiberensis*, so the related metabolic pathways and products might be extremely similar. In potato, the protein sequences and conserved domains of *HMGR*, *CAS*, and *SQS* were more dissimilar and *SMT1* had little similarity with *D*. *composita*—the gene sequences had no similarity with *D*. *composita*. This means that *HMGR*, *CAS*, and *SQS* had some structural similarity in *D*. *composita* and potato, but the products in potato might involve carotenoids or other lipids rather than steroidal sapogenins [29]. The structure and function of *SMT1* in potato were different from that in *D*. *composita*, especially in the Sterol_MT_C domain. *SMT1* had another conserved domain which used adenosylmethionine as a substrate for methyl transfer. Thus, it might catalyze the methionine methyl transference instead of the sterol in potato.

### Expression of four specific genes

**Fig 7:**
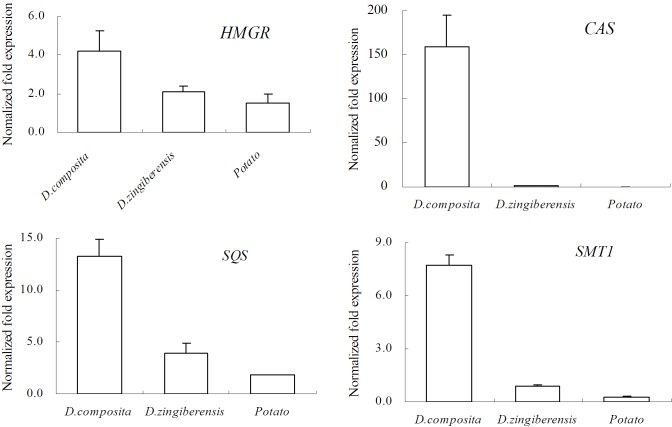
Expression of four transcripts related to steroidal sapogenin in the tuber of *D*. *composita*, *D*. *zingiberensis*, and potato. The *D*. *composita* and *D*. *zingiberensis* samples came from 18-month-old plants, and the samples of potato from 6-month-old plants.

The expression of 4 genes related to steroidal sapogenin biosynthesis in the tuber of *D*. *composita*, *D*. *zingiberensis*, and potato was analyzed by relative quantitative RT-PCR. The results are shown in Fig 7. The expression of *HMGR*, *CAS*, *SQS*, and *SMT1* in *D*. *composita* was found to be 2.0, 159.1, 3.4, and 8.9 times that in *D*. *zingiberensis*, respectively. Therefore, steroidal sapogenin biosynthesis was more rapid and efficient in *D*. *composita*. The natural role of steroidal sapogenins is to confer protection against potential pathogens [9]. It is thought it might play a part in defense-related signaling processes in the presence of pathogen infection [30]. *D*. *zingiberensis* has been cultivated as a medicine in favorable planting conditions for several decades, so the capacity of its defense response might have degenerated without frequent pathogen accumulation. Conversely, *D*. *composita* has been living in the wild and has better maintained its defense capability.

The expressions of *HMGR*, *SQS*, and *SMT1* in *D*. *composita* were 2.8, 7.3, and 27.9 times greater than in potato, respectively. *CAS* was hardly expressed in potato at all. There were no steroidal sapogenins synthesized in potato because it lacked a key enzyme in its metabolic pathway. In fact, all the data related to *CAS* in potato in the public domain had been predicted though genome sequencing [31]. *CAS* might exist as a non-functional gene in potato. Evolution, maybe, was one of the important reasons for this difference (different genera of plants producing different antimicrobial compounds to resist different environments in the course of evolution).

## Conclusions

Steroidal sapogenins are generally considered to be part of the defense system in plants. They have recently been attracting some interests due to their wide spectrum of biological, pharmacological, economical, and medicinal importance. Some species of Dioscoreaceae, like *D*. *zingiberensis*, are utilized mainly as a material to extract steroidal sapogenins for the medicinal industry [5]. As a novel resource, the quantity and quality of steroidal sapogenins in *D*. *composita* are exceptional. Determination of the genes involved in this secondary metabolism should allow researchers to improve the production of its effective constituents by the various techniques of molecular biology and metabolic regulation. Despite the recent great improvements in sequencing technology, no genomic data were available for consultation for any of the Dioscoreaceae species.

In this work, we generated 27,720 unigenes through transcriptome sequencing, and predicted a pathway for steroidal sapogenin backbone biosynthesis in *D*. *composita*. Of these unigenes, 531 were obtained that relate to secondary metabolism, of which, 79 unigenes were associated with steroidal sapogenin biosynthesis. There were 21 steps in the pathway from acetyl-CoA to cycloartenol, the precursor of steroidal sapogenin biosynthesis [32]. A variety of steroidal sapogenins were produced through complex modification of cycloartenol, for example, the catalysis of stereospecific P450 cytochromes, but this process was unclear [33]. The expression profiles of 10 related transcripts revealed that all the organs involved in this pathway, gene expression was the largest in the tubers and leaves. This was because the products of this pathway included membrane and defense constituents. The low expression observed in shoots suggested that the secondary metabolism was not prevalent at the seedling stage.

Through a comparison with *D*. *zingiberensis*, the expression of 4 related genes were found to be the greater in *D*. *composita*, and result in greater enrichment of steroidal sapogenins in *D*. *composita*. This may be caused by the stronger defense response in *D*. *composita*. No expression of key enzymes led to a lack of steroidal sapogenins in potato. This preliminary information allows us to understand the biosynthetic pathway of steroidal sapogenins in *D*. *composita*, and should serve as a foundation for manipulating the secondary metabolism to produce health-promoting ingredients. In addition, the transcriptome data we have derived constitutes a much more abundant genetic resource that can be utilized to benefit further in-depth studies on *D*. *composita*.

## Material and Methods

### Plant material

No specific permissions were required for the field studies in Shaoguan, Guangdong Province, China. It was, however, confirmed that these studies did not involve endangered or protected species.

The GPS coordinates of the location of the field studies is 24.305984559949003, 113.96962703698728. *D*. *composita* samples were collected from 18-month-old plants and 1-month-old shoots (counted from field planting) that were cultivated in Shaoguan. For the 18-month-old plants, leaves, stems, and tubers were separately harvested from six plant individuals. Samples were cut and mixed using samples from the top, middle, and bottom parts of the same tissues from an individual. For the 1-month-old shoots, six whole plants were collected. All samples were immediately frozen in liquid nitrogen after cleaning with germ-free water, and stored at—80°C before use.

*D*. *zingiberensis* tuber samples were collected from 18-month-old plants, Chinese yam tuber samples were collected from 9-month-old plants, potato tuber samples were collected from 6-month-old plants, and cassava tuber samples were collected from 12-month-old plants. All of these were cultivated in Shaoguan. For each kind of plant, samples were separately harvested from six individuals, cut, and the samples from the top, middle, and bottom parts of the tuber from each individual mixed.

### RNA isolation and sequencing

Total RNA was isolated by using a TIANDZ Column Plant RNAout 2.0 (with DNase) from various organs and development stages, respectively. The quality and concentration of RNA were detected by electrophoresis and a Backman Counter (DU730). Parts of the RNA from different organs (18-month-old plants) were mixed together in equal measure and sent to NOVOGENE (Beijing, China) where the cDNA library was established and Illumina sequencing was completed.

The remaining RNA from different tissues (18-month-old plants), RNA extracted from 1-month-old shoots, and RNA from different plants were used for real-time PCR analysis. About 1 μg of total RNA was reverse transcribed into single-stranded cDNA using a TaKaRa PrimeScript RT reagent kit with gDNA Eraser (Perfect Real Time) for each sample. The cDNA solutions were diluted 10 times for templates in real-time PCR.

### Steroidal sapogenin determination

Freeze-dried powdered tuber (1 g) was hydrolyzed with hydrochloric acid (2 M) by boiling in a water bath for 4 h. This was filtered and the residue washed to neutral. It was then dried at 80°C. The residue was put into a soxhlet extractor with filter paper package, refluxed with petroleum ether (80°C) for 4 h. The petroleum ether was eevaporated and the residue dissolved in chloroform, diluting it to 10 mL.

Steroidal sapogenin extractions were measured using capillary gas chromatography (GC7890II) equipped with a TM-5 Stainless steel capillary column (30 m × 0.20 mm × 0.25 μm) and a flame ionization detector. Other parameters include: column temperature: 270°C; column head pressure: 123 kPa; vaporizer temperature: 330°C; detector temperature: 310°C; N_2_ flow rate (carrier gas): 2.0 mL/min; split ratio: 40:1; volume injected: 10 μL. Standard sapogenin (purchased from Fluka) was dissolved in chloroform.

### Real-time PCR and expression analysis

The primers of 12 selected transcripts were designed online using Primer-NCBI. The quantitative reactions were finished with TaKaRa SYBR Premix ExTaq II (TliRNaseH Plus) on Eppendorf Mastercycler Real-Time Thermal Cylers. PCR conditions: 95°C for 2 min, followed by 40 cycles of 95°C for 15 s, 60°C for 15 s, and 72°C for 20 s. The expressions of selected genes were normalized against glyceraldehyde-3-phosphate dehydrogenase (GAPDH) which was an internal reference gene. Moreover, the relative expression was counted using the 2^–ΔΔCt^ method. All real-time PCR experiments were repeated using three biological, and three technical, replications.

### Accession of data

The raw data have been submitted to Sequence Read Archive of NCBI with accession numbers: SRR1525775, SRR1525776, SRR1525777, SRR1525779, SRR1525790, SRR1525791, SRR1525792, and SRR1525793.

### Software

Trinity (v2012-10-15), http://sourceforge.net/projects/trinitynaseq/files/

Blastx (v2.2.27+), ftp://ftp.ncbi.nlm.nih.gov/blast/executables/LATEST/

Hmmscan (HMMER 3.0), http://hmmer.janelia.org/software/

KAAS (r140224), http://www.genome.jp/tools/kass/

## Supporting Information

S1 Table Annotations against NR. (XLSX)

S2 Table Annotations against Arabidopsis protein database. (XLSX)

S3 Table Annotations against Rice protein database. (XLSX)

S4 Table Annotations against KEGG. (XLS)

S5 Table Annotations against Pfam. (XLS)

S6 Table Putative unigenes related to steroidal sapogenin biosynthesis. (XLSX)
